# The relationship between women's personality traits and fear of childbirth, birth satisfaction, and postpartum depression

**DOI:** 10.1590/1806-9282.20231730

**Published:** 2024-08-16

**Authors:** Feyza Aktaş Reyhan, Elif Dağlı

**Affiliations:** 1 Kütahya University of Health Sciences, Faculty of Health Sciences, Department of Midwifery - Kütahya, Turkey.; 2 Çukurova University, Abdi Sütcü Vocational School of Health Services, Department of Health Care Services - Adana, Turkey.

**Keywords:** Woman, Personality traits, Fear, Parturition, Patient satisfaction, Postpartum depression

## Abstract

**OBJECTIVE:**

This study was conducted to determine the relationship between women's personality traits and their fear of childbirth, birth satisfaction, and postpartum depression.

**METHODS:**

This cross-sectional study was conducted between April and August 2022 among healthy third-trimester pregnant women aged 18-49 years who applied to the obstetrics and gynecology outpatient clinic of a state hospital. Data were collected by the researchers by face-to-face interview method in three stages. Participants were administered the Personal Information Form, the Five-Factor Personality Scale, and the Birth Anticipation/Experience Scale at the first interview; the Birth Satisfaction Scale on the 10th day after normal birth; and the Edinburg Postpartum Depression Scale 4 weeks after birth.

**RESULTS:**

There was a significant positive correlation between neurotic personality traits and fear of childbirth and postpartum depression, while there was a negative correlation with other personality traits (p<0.001). There was no significant relationship between birth satisfaction and personality traits (p>0.05). The effect of personality traits on fear of childbirth and postpartum depression was analyzed by multiple linear regression analysis. The regression model tested for the effect of personality traits on fear of childbirth and postpartum depression was found significant (p<0.001). According to the model, 26% of the variability in fear of childbirth and 9.1% of the variability in postpartum depression were explained by personality traits.

**CONCLUSION:**

This study showed that neuroticism, which is one of the personality traits of women, had a positive effect on fear of childbirth and postpartum depression. No significant relationship was found between birth satisfaction and personality traits.

## INTRODUCTION

Childbirth, which is a physiological process, is the most special and meaningful moment for women, but it is a process that every woman experiences differently. Although the literature shows that fear of childbirth is associated with negative birth satisfaction and postpartum depression, it may also cause many problems such as pregnancy and birth complications, difficulties in mother-infant attachment, and cesarean section^1,2^. It is mentioned that fear of childbirth, the etiology of which is based on many factors such as age, education, income level, lack of information, fear of intervention, negative birth stories, and previous traumatic experiences^3^, is closely related to the personality traits of women^4^.

Personality traits of women are considered to be an important factor, especially in perceiving stressful events and developing coping strategies^4^. The Five-Factor Model, which is frequently used to assess personality traits, defines five dimensions of personality: extraversion (*sociable, sociable*, *positive emotions*), openness to experience (*curious, creative*, *questioning*), agreeableness (*trustworthy, obedient*, *compassionate*), conscientiousness (*hardworking, planned*, *determined*), and neuroticism (*anxiety, depression*, *pessimistic, angry*). Personality traits, particularly neurotic and extraverted personality, have been reported to be associated with health outcomes^5^. Likewise, it is observed that women's personality traits have an impact on their feelings, thoughts, expectations, and experiences during pregnancy, birth, and postpartum periods^6^. It has been reported that women with certain distinct personalities experience severe fear of childbirth and experience the birth and postpartum periods as traumatic^7^. In particular, neurotic personality structure may contribute to women's dissatisfaction with their birth experiences. Studies have shown that women with neurotic traits are less likely to have a positive birth experience^7^ and are at risk for postpartum depression^8^.

Fear of childbirth, birth satisfaction, and postpartum depression are interrelated and important issues in terms of maternal, infant, family, and community health as well as the quality of health care. Being aware of the effect of personality traits on these issues may enable early interventions to be made. Therefore, it is thought that determining the personality traits of women in the antenatal period may contribute to the provision of care and counseling in pregnancy, birth, and postpartum processes. There are a limited number of studies in the literature on the relationship between personality traits and fear of childbirth, labor satisfaction, and postpartum depression. This study was conducted to determine the relationship between women's personality traits and their fear of childbirth, birth satisfaction, and postpartum depression.

## METHODS

### Study design

This is a descriptive and correlational study.

### Participants

The study was conducted between April and August 2022 in the obstetrics and gynecology outpatient clinic of a state hospital in southern Turkey. The population of the research consisted of 24,828 pregnant women who came to the delivery clinic of a hospital. In sample selection, the sample calculation formula (95% confidence interval, 5% margin of error, 50% incidence) was used. According to the calculation result, the sample size was determined as 358. Initially, 556 participants were interviewed, but 34 of them underwent cesarean section and 24 of them did not volunteer to participate in the next phase of the study. The study was completed with a total of 498 women. Inclusion criteria in the study were (1) being between the ages of 18-49 years (reproductive age), (2) being in the third trimester of pregnancy (28-40 weeks), (3) planning a vaginal birth,(4) avoiding communication problems, and (5) volunteering to participate in the research. Exclusion criteria were (1) experiencing complications during pregnancy, (2) failure of vaginal delivery, and (3) postpartum complications.

### Data collection tools

Personal Information Form, Five-Factor Personality Scale, Wijma Birth Anticipation/Experience Scale, Birth Satisfaction Scale, and Edinburg Postpartum Depression Scale were used as data collection tools. The personal information form includes questions about the socio-demographic characteristics of the women.

The Five-Factor Personality Scale (BFKO) developed by Rammstedt and John is used to determine the personality traits of individuals^9^. The scale consists of 10 items on a 5-point Likert scale. BFKO consists of five factors: extraversion, agreeableness, conscientiousness, openness to experience, and emotional instability. Wijma Birth Anticipation/Experience Scale (W-DEQ), adapted into Turkish by Korukcu et al., is used to measure the fear of childbirth experienced by women^10^. The scale consists of 33 items and is a 6-point Likert-type scale. A high total score indicates a high level of fear.

Scale for Assessing Maternal Satisfaction in Normal Delivery (NDAMDÖ) was developed by Gungor and Beji to evaluate the experiences of mothers during labor and early postpartum period in the hospital^11^. As the total score obtained from the scale increases, the mother's satisfaction with the care she received in the hospital during vaginal delivery also increases. The Edinburgh Postnatal Depression Scale (EDSDO) was developed by Cox et al. to determine the depression and risks of women in the postpartum period^12^. The scale is a 10-item, 4-point Likert-type self-report scale.

### Procedure

Data were collected by a single researcher by conducting face-to-face interviews at the hospital. The study was conducted in three stages. In the first step, the Personal Information Form, Five-Factor Personality Scale, and Birth Anticipation/Experience Scale were applied to pregnant women who applied to the obstetrics and gynecology outpatient clinic and agreed to participate. They were informed about the other steps of the study and their contact information was obtained. In the second stage, women who participated in the first study, had a normal delivery, and were in the first 10 days postpartum were called, and the Birth Satisfaction Scale was applied. In the last stage, women who completed at least 4 weeks postpartum were contacted by phone and evaluated with the Edinburg Postpartum Depression Scale. The administration of the questionnaires took an average of 25 min.

### Statistical analysis

Data were analyzed in the IBM SPSS Statistics 26.0 program. The Pearson correlation method was used in the relationship between scale scores and personality traits. In addition, the effect of personality traits on postpartum depression level and fear of childbirth was examined by the multiple linear regression analysis method. For comparison by groups, independent-sample t-test and ANOVA were used. A significance level of p<0.05 was used for statistical analysis.

### Ethical procedure

Ethics committee approval was received for the research by the decision of the Cukurova University Non-Interventional Clinical Research Ethics Committee (Date: April 02, 2022; Decision no: 121/84). Participants were informed about the purpose of the study and their consent was obtained. Data were collected from women who voluntarily agreed to participate in the study. The study was conducted in alignment with the Principles of the Declaration of Helsinki.

## RESULTS

Of the 498 participants in the study, 91.2% were under 35 years of age, 36.7% had secondary education, 50% were employed, and 49.6% lived in the province. Notably, 64.1% of the women were multiparous and 71.7% had planned pregnancies. There was no difference between the fear of childbirth, postpartum satisfaction, and postpartum depression scores of the women according to their age, educational level, place of residence, and number of births (p>0.05). The mean score comparisons of the scales according to the sociodemographic characteristics of the participants are presented in detail in Table 1.

**Table 1:** Distribution of women according to demographic variables and comparison table between fear of childbirth, postpartum satisfaction, and postpartum depression levels according to these variables.

Variable		f (%)	Fear of childbirth	Birth satisfaction	Postpartum depression
			Mean (SD)	Mean (SD)	Mean (SD)
Age** (years)					
	Under 35	454 (91.2)	29.77 (0.19)	182.78 (25.31)	11.94 (4.01)
	35 and above	44 (8.8)	29.8 (10.21)	185.82 (27.12)	12.5 (4.38)
	p		0.986	0.451	0.384
Education level***					
	Literate	63 (12.7)	28.92 (8.13)	185.21 (23.34)	12.43 (4.03)
	Primary education	155 (31.1)	29.79 (10.86)	185.43 (26.12)	11.62 (3.82)
	Secondary education	183 (36.7)	30.49 (10.32)	181.03 (25.88)	12.17 (4.06)
	High education	97 (19.5)	28.93 (10.06)	181.67 (24.84)	11.98 (4.34)
	p		0.572	0.357	0.493
Employment status**					
	Yes	249 (50)	31.05 (10.75)	180.12 (25.37)	12.08 (3.93)
	No	249 (50)	28.49 (9.44)	185.98 (25.25)	11.9 (4.15)
	p		0.005*	0.01*	0.618
Place of residence***					
	Province	247 (49.6)	30 (9.62)	182.68 (25.66)	11.89 (3.94)
	County	175 (35.1)	29.76 (11.1)	184.69 (25.07)	12.35 (4.07)
	Village	76 (15.3)	29.05 (9.89)	180.49 (25.75)	11.51 (4.25)
	p		0.666	0.321	0.699
Total number of births**					
	Primiparous	179 (35.9)	30.03 (9.88)	184.6 (25.85)	11.68 (3.98)
	Multiparous	319 (64.1)	29.62 (10.36)	182.18 (25.23)	12.17 (4.06)
	p		0.671	0.31	0.188
Pregnancy being planned**					
	Yes	357 (71.7)	30.34 (10.69)	182.99 (25.81)	12.04 (4.09)
	No	141 (28.3)	28.31 (8.66)	183.21 (24.62)	11.87 (3.91)
	p		0.045*	0.932	0.655
Getting adequate midwife support during birth**					
	Yes	384 (77.1)	29.87 (10.12)	183.47 (25.95)	12.22 (4.03)
	No	114 (22.9)	29.44 (10.44)	181.65 (23.78)	11.25 (3.99)
	p		0.694	0.504	0.024*

* p<0.05

** independent groups t-test

*** ANOVA test.

Descriptive statistics for the total scores of fear of childbirth, birth satisfaction, and postpartum depression and the sub-dimension scores of personality traits are shown in Table 2. Scores for all personality traits ranged between 2 and 10, with the highest mean being openness to experience (X=5.92) and the lowest mean being neuroticism (X=4.76) (Table 2).

**Table 2:** Descriptive statistics related to personality traits and total scores of fear of childbirth, birth satisfaction, and postpartum depression.

Score	Min	Max	Mean (SD)	Skewness	Kurtosis
Fear of childbirth	8	70	29.77 (10.19)	1.096	1.915
Birth satisfaction	110	215	183.05 (25.46)	-0.294	-0.812
Postpartum depression	0	20	11.99 (4.04)	0.239	-0.348
Extroversion	2	10	5.82 (2.61)	-0.09	-1.386
Agreeableness	2	10	5.81 (2.6)	-0.08	-1.378
Conscientiousness	2	10	5.81 (2.6)	-0.077	-1.372
Openness to experience	2	10	5.92 (2.62)	-0.147	-1.374
Neuroticism	2	10	4.76 (2.78)	0.678	-0.93

In the study, the relationship between women's personality traits and fear of birth, birth satisfaction, and postpartum depression levels was examined. There is a moderate relationship between fear of childbirth and extroversion (r=-0.414), agreeableness (-0.413), conscientiousness (r=-0.416) scores, and a low and negative relationship between openness to experience scores (r=-0.354) (p<0.001). There was a positive and moderately significant relationship between fear of childbirth and neuroticism (r=0.469; p<0.001). There is a low and negative relationship between postpartum depression and personality traits of extroversion (r=-0.202), agreeableness (r=-0.198), conscientiousness (r=-0.199), and openness to experience (r=-0.213) (p<0.001). There was a positive and low-level significant relationship between postpartum depression and neuroticism (p<0.001). There is no significant relationship between birth satisfaction and personality traits (p>0.05).

The effect of personality traits on fear of childbirth and postpartum depression was examined with the multiple linear regression analysis method (Table 3). The regression model tested for the effect of personality traits on fear of childbirth was found to be significant (F=34.52, p<0.001). According to the model, 26% of the variability in fear of childbirth is explained by personality traits. Neuroticism and conscientiousness were found to be significant predictors of fear of childbirth in women.

**Table 3:** Regression model testing the effect of personality traits on fear of childbirth and postpartum depression.

Independent variable	Fear of childbirth		Postpartum depression	
	B	p	β	p
Extroversion	-0.207	0.748	-0.616	0.389
Agreeableness	0.208	0.761	0.953	0.207
Conscientiousness	-0.701	<0.001	-0.057	0.931
Openness to experience	0.137	0.725	-0.301	0.057
Neuroticism	0.337	<0.001	0.279	<0.001
Model statistics	F=34.52; p<0.001		F=9.901; p<0.001	
	R^2^=0.26		R^2^=0.091	

The regression model tested for the effect of personality traits on postpartum depression was found to be significant (F=34.52, p<0.05). According to the model, 9.1% of the variability in postpartum depression is explained by personality traits. Among the personality traits, only neuroticism was a significant predictor of postpartum depression in women and its effect was found to be positive (r=0.285; p<0.001).

## DISCUSSION

This study was conducted to determine the relationship between women's personality traits and their fear of childbirth, birth satisfaction, and postpartum depression. In this study, it was found that extraversion, agreeableness, conscientiousness, and openness to experience had a negative effect on fear of childbirth and postpartum depression, while neuroticism had a positive effect. No significant relationship was found between personality traits and birth satisfaction. In the limited number of studies in the literature, it has been reported that women's personality traits are a variable that may affect fear of childbirth^13-15^. Dursun et al. found that there was a relationship between personality traits and fear of childbirth; it was found that fear of childbirth was higher in pregnant women with neurotic and psychotic personalities, while fear of childbirth was lower in pregnant women with extroverted personality^15^. Conrad and Trachtenberg reported significant correlations between personality traits and fear of childbirth and birth experience; neuroticism was associated with birth experiences^6^. It is observed that our research findings are in parallel with the literature. In addition, the regression model tested according to the effect of women's personality traits on fear of childbirth was significant, and neuroticism and conscientiousness were found to be significant predictors of fear of childbirth. While neuroticism has a positive effect on fear of childbirth, conscientiousness has a negative effect. Similarly, Uludağ et al. used personality traits to predict fear of childbirth and found that pregnant women with extroverted personality traits were significantly less likely to experience fear of childbirth and those with personality traits close to neuroticism had a high level of fear of childbirth^14^. The characteristics of individuals with neurotic personality such as being anxious and agitated suggest that women with this personality increase the risk of experiencing fear of childbirth. It can be said that extroverted individuals can cope with the fear of childbirth by adapting to the pregnancy process more easily with their positive outlook on life and their ability to cope with the difficulties they face.

In this study, personality traits were found to be an effective variable on postpartum depression. When the literature was examined, it was found that personality traits of women did not have a direct effect on postpartum depression^16^, while some studies found that personality traits were associated with psychological and mental disorders^8,17^. Studies have shown that there is a significant positive correlation between postpartum depression and neuroticism, while there is a significant negative correlation with other personality traits^17,18^. Sunay et al. found a significant negative correlation between postpartum depression and extraversion, conscientiousness, emotional stability, and agreeableness. The multiple linear regression model showed that personality traits such as conscientiousness, emotional stability, and mildness are important determinants of postpartum depression^17^. These results support our current research findings. According to our regression model, 9.1% of the variability in postpartum depression is explained by personality traits. Neuroticism is a significant predictor of postpartum depression in women and its effect was found to be positive. Marín-Morales et al. found that postpartum depression had a predictive effect on extraversion and agreeableness^19^. Although our results cannot be generalized to all women, it is important to evaluate personality traits along with other risk factors in order to identify women at risk of postpartum depression at an early stage.

In this study, no significant relationship was found between birth satisfaction and personality traits. However, the results of a few studies in the literature report that agreeableness and conscientiousness are associated with higher birth satisfaction, while neuroticism is associated with lower satisfaction^20,21^. It would be useful to consider the personality characteristics of women in the evaluation of birth satisfaction, which is an important parameter for maternal, infant, and family health, and to provide care and support accordingly.

## LIMITATIONS

The fact that the study was conducted with women who applied to the obstetrics and gynecology outpatient clinics of a hospital in Turkey limits the generalization of the results to other health centers and other regions of the country.

## CONCLUSION

In this study, it was determined that neuroticism had a positive effect. No significant relationship was found between birth satisfaction and personality traits. Fear of childbirth, birth satisfaction, and postpartum depression are interrelated and important issues for improving maternal, infant, and family health. Women's personality traits should be evaluated at an early stage, the negative role of neuroticism should be recognized, and necessary care and counseling should be provided.

## ETHICS APPROVAL

Ethics approval was obtained from the Ethics Committee of Çukurova University Medical Faculty (dated April 8, 2022, numbered 121/84).
